# Supplementary material for: Patient safety culture in public hospitals of Ethiopia: A systematic review and meta-analysis
Source: PLoS One. 2025 Jun 4;20(6):e0325723. doi: 10.1371/journal.pone.0325723 (PMC12136323; doi:10.1371/journal.pone.0325723)
Supplement: Supplementary file 2 — (DOCX) [file pone.0325723.s002.docx]

Supplementary file 2: Methodological quality assessment of 14 included studies using the Newcastle-Ottawa quality assessment scale

| Study | Selection | | | | Comparability | Outcome | |  |
| --- | --- | --- | --- | --- | --- | --- | --- | --- |
|  | Representativeness of the sample | Sample size | Non respondents | Ascertainment of the exposure (maximum score=2) | The subjects in different outcome groups are comparable, based on the study design or analysis. Confounding factors are controlled (maximum score=2) | Assessment of the outcome (maximum score=2) | Statistical test | Total (10) |
| Mohamed, F. et al (2) | 1 | 1 | 1 | 1 | 1 | 2 | 1 | 8 |
| Wami, SD. et al (30) | 1 | 1 | 1 | 1 | 2 | 2 | 1 | 9 |
| Kumbi, M. et al (31) | 1 | 1 | 1 | 1 | 1 | 2 | 1 | 8 |
| Ayisa, A. et al (27) | 1 | 1 | 1 | 1 | 1 | 2 | 1 | 7 |
| Mekonnen, AB. et al (22) | 1 | 1 | 1 | 1 | 1 | 2 | 1 | 8 |
| Garuma, M. et al (21) | 1 | 1 | 1 | 1 | 1 | 2 | 1 | 8 |
| Mitiku M et al (35) | 1 | 0 | 1 | 1 | 1 | 2 | 1 | 7 |
| Yismaw W et al (32) | 1 | 1 | 0 | 1 | 1 | 1 | 1 | 6 |
| Ayanaw T et al (28) | 1 | 1 | 1 | 1 | 1 | 1 | 1 | 7 |
| Shashamo BB et al (34) | 1 | 1 |  | 1 | 1 | 1 | 1 | 7 |
| Yayehrad T et al (19) | 1 | 1 | 1 | 1 | 1 | 2 | 1 | 8 |
| Ali M et al (29) | 1 | 1 | 1 | 1 | 1 | 2 | 1 | 8 |
| Mulugeta TT (33) | 1 | 1 | 0 | 1 | 1 | 1 | 1 | 6 |
| Atinafu D et al (18) | 1 | 1 | 1 | 1 | 2 | 2 | 1 | 9 |
