# Supplementary material for: Patient safety culture in public hospitals of Ethiopia: A systematic review and meta-analysis
Source: PLoS One. 2025 Jun 4;20(6):e0325723. doi: 10.1371/journal.pone.0325723 (PMC12136323; doi:10.1371/journal.pone.0325723)
Supplement: Attachment — Submitted filename: Comment Patient safety.docx [file pone.0325723.s005.docx]

- **Clarity of Objectives:**

They lack clear delineation, making it less accessible to readers.

- **Abstract:**

The abstract is comprehensive but dense. Key details such as the number of studies, methodology, and major findings could be presented more concisely.

- **Consistency:**

Phrases like "patient safety culture" and "culture of patient safety" are used interchangeably, potentially confusing readers.

- **Background:**

The introduction provides extensive global context but offers limited background on the Ethiopian healthcare system's specific challenges related to patient safety.

- **Quality Assessment:**

While the manuscript mentions the use of the Newcastle-Ottawa Scale, it does not detail the quality ratings or how low-quality studies impacted the results.

- **Heterogeneity and Publication Bias:**

The study reports high heterogeneity (I² = 93.9%), which is expected given the variability in healthcare settings across Ethiopia. However, the implications of such heterogeneity on the reliability of pooled estimates are not discussed.

- **Implications and Recommendations:**

Recommendations are general (education, training, guidelines) and lack specificity regarding implementation within Ethiopia’s resource constraints.

- **Language and Grammar:**

Grammatical errors and awkward phrasing detract from the manuscript's professionalism. For example:

- *"Due to unsafe care, 134 million adverse events occur in hospitals in LMICs each year resulting in 2.6 million deaths"* could be rephrased for clarity. Conduct a thorough language review or use professional editing services to polish the manuscript.
- **Results**
  The narrative could benefit from restructuring for clarity. For example:
  - Group statistical findings (e.g., prevalence, heterogeneity) separately from qualitative interpretations.
  - Add a clear flow to the explanation of subgroup and sensitivity analyses.
- **Addressing Heterogeneity**:

Significant heterogeneity (I² = 93.0%) remains a limitation, and while the random-effects model mitigates this, further exploration into the sources of heterogeneity would strengthen the paper. Were differences in survey methodology, sample size, or health system infrastructure contributors?

- **Discussion**:

While the comparison to other countries is helpful, the discussion could elaborate on the root causes of Ethiopia's low patient safety culture prevalence (e.g., resource constraints, health system gaps, or cultural attitudes toward patient safety). The implications for practice should specify who will lead the recommended changes (e.g., government agencies, hospital administrators) and what key steps are needed for implementation. Ensure that all references are cited in alignment with journal requirements. Some critical comparisons (e.g., studies from Oman, Taiwan, and South Africa) could include more detailed descriptions of how those countries addressed patient safety culture gaps.

- **Limitations Section**:

The discussion of limitations is brief. Consider addressing potential publication bias (despite Egger’s test) and the challenges of using self-reported measures like the HSOPSC, which may introduce social desirability bias.
